# Association between inflammatory markers and serum paraoxonase and arylesterase activities in the general population: a cross-sectional study

**DOI:** 10.1186/s12944-021-01508-7

**Published:** 2021-07-31

**Authors:** Christa Meisinger, Dennis Freuer, Achim Bub, Jakob Linseisen

**Affiliations:** 1 Chair of Epidemiology, University of Augsburg, University Hospital Augsburg, Stenglinstr. 2, 86156 Augsburg, Germany; 2 Independent Research Group Clinical Epidemiology, Helmholtz Zentrum München, German Research Centre for Environmental Health, 85764 Neuherberg, Germany; 3 Department of Physiology and Biochemistry of Nutrition, Max-Rubner-Institut, Federal Research Institute of Nutrition and Food, 76131 Karlsruhe, Germany

**Keywords:** Inflammation, PON1, Arylesterase activity, Paraoxonase activity, Oxidative stress

## Abstract

**Background:**

Recent studies focused on modulating factors of paraoxonase-1 (PON1) activity. In some studies the association between pro-inflammatory markers and PON1 activity was examined, but so far no population-based investigations on this issue have been conducted. The present study investigated the relationships between the pro-inflammatory markers tumor necrosis factor (TNF)-α, leptin, interleukin (IL)-6, and high-sensitive C-reactive protein (hs-CRP) and paraoxonase and arylesterase, two hydrolytic activities of PON1, in the population-based Bavarian Food Consumption Survey II.

**Methods:**

Based on 504 participants (217 men, 287 women), the relationship between the pro-inflammatory markers and the outcomes paraoxonase and arylesterase activities were investigated using multivariable linear models.

**Results:**

Circulating plasma levels of leptin (*P*-value < 0.0001), hs-CRP (*P*-value = 0.031) and IL-6 (*P*-value = 0.045) were significantly non-linearly associated with arylesterase activity. Leptin levels were also significantly associated with paraoxonase activity (*P*-value = 0.024) independently from confounding factors, including high-density lipoprotein (HDL) cholesterol. With increasing levels of these inflammatory parameters, arylesterase and paraoxonase activities increased; however, at higher levels (> 75th percentile) the activities reached a plateau or even decreased somewhat. After Bonferroni-Holm correction, only leptin remained non-linearly but significantly associated with arylesterase activity (adjusted overall *P*-value < 0.0001). Neither age nor sex nor obesity modified the associations. No association was found between TNF-α and paraoxonase or arylesterase activity.

**Conclusions:**

The present findings suggest that in persons with very high levels of inflammation, PON1 activity may be impaired, a fact that might subsequently be accompanied by a higher risk for cardiometabolic diseases. Whether or not the measurement of PON1 activity in combination with a lipid profile and certain inflammatory markers could improve the prediction of cardiometabolic diseases in middle-aged individuals from the general population should be evaluated in clinical studies.

## Background

Nowadays one of the greatest challenges of medicine is the increasing prevalence of cardiometabolic diseases, such as type 2 diabetes, non-alcoholic fatty liver disease, and metabolic syndrome [1]. This increase is mainly due to overnutrition in combination with physical inactivity and sedentarism [2]. In the past two decades, the pathophysiological mechanisms that contribute to the manifestation of cardiometabolic diseases, which are commonly associated with premature death, have been the subject of numerous research activities [3, 4]. A wealth of evidence highlights chronic low-grade inflammation and oxidative stress as common underlying mechanisms. A large number of adipokines [5] which are mainly secreted from adipose tissue, seem to be involved in the regulation of energy expenditure, carbohydrate and lipid metabolism, hunger/satiety, immune response, inflammation and oxidative stress [6, 7]. Evidence suggests that the mechanism by which inflammation and oxidative stress are involved in the development of cardiometabolic diseases is multifactorial. However, it seems that changes in high-density lipoprotein (HDL) structure and function during inflammation could play a role [8]. HDL cholesterol is known to correlate inversely with atherosclerotic diseases. Its protective effect is due to its ability to enhance cholesterol efflux from peripheral cells back to the liver. However, a number of additional protective properties of HDL cholesterol have recently been discovered, such as anti-inflammatory and antioxidant functions [9]. One of the anti-atherogenic effects of HDL cholesterol is due to paraoxonase-1 (PON1), a glycoprotein that is synthesized primarily by the liver and is mostly bound on HDL in the blood [10]. PON1 is endowed with three hydrolytic activities, namely paraoxonase activity, arylesterase activity, and lactonase activity [10]. Recent research activities thus focus on modulating factors of PON1 activity [11–13]. A few prior studies investigated whether adipokines had an effect on PON1 activity, but these studies were mainly based on patient groups with a certain disease [12, 14, 15], on females or males only [11, 13], or on small sample sizes [16]. So far, data in a large population-based study including healthy individuals with mainly low values of inflammatory markers are still scarce. The present study can contribute to closing this research gap by investigating the association between the pro-inflammatory factors tumor necrosis factor (TNF)-α, leptin, interleukin (IL)-6, and high-sensitive C-reactive protein (hs-CRP) and paraoxonase activity as well as arylesterase activity in a sample of the general adult population in Germany. Furthermore, it was examined whether the associations were modified by obesity, sex or age.

## Methods

The Bavarian Food Consumption Survey II (BVSII) is a representative cross-sectional study of the German-speaking population of Bavaria, Germany, and was conducted in 2002–2003. The examination included the conduct of a personal interview, the assessment of three 24-h dietary recalls by telephone, the collection of blood and the measurement of height, weight and waist circumference. Altogether 1050 randomly selected individuals aged 13 to 80 years participated in the personal interview, i.e. 71% of the eligible individuals participated in the study. Altogether, 879 completed the baseline interview and at least one dietary recall and were thus invited to a study center for blood collection and anthropometric measurements; 568 participants (65%) completed the requested examinations. Detailed information on the BVSII has been described in detail elsewhere [17–19]. Written informed consent was provided by all study participants and the study was approved by the ethics committee of the Bavarian Medical Association on June 19th, 2002 (Ethik-Kommission Nr. 02111). The investigations were carried out in accordance with the Declaration of Helsinki. After exclusion of 64 participants with missing values in any of the relevant variables, the present study included 504 BVSII participants for analysis.

### Laboratory methods

Venous serum and EDTA blood samples were obtained in a non-fasting state. After processing and aliquoting, the samples were stored at − 80 °C until analysis. Paraoxonase and arylesterase activities were spectrophotometrically measured in serum samples at a temperature of 25 °C and a pH of 8 [20]. Using paraoxon as the substrate, the enzymatic activity of PON1 was determined; the reaction was recorded at 405 nm. One unit of paraoxonase activity equals 1 nmol of *P*-nitrophenol formed/min/ml. Arylesterase activity was determined at 270 nm with phenylacetate as the substrate and one unit of arylesterase activity equals 1 μmol of phenylacetate hydrolysed/min per ml [20].

The inflammation markers hs-CRP, IL-6, and TNF-α were measured in plasma using commercial ELISAs (Biosource, Brussels, Belgium). The methods have been described in detail elsewhere [21]. All assays had an intra-assay coefficient of variation (CV) < 7%, and an interassay CV < 9% [21]. The polypeptide leptin was measured using a radioimmunoassay [22].

### Statistical analysis

All associations between the exposures TNF-α, leptin, IL-6 and hs-CRP and the outcomes paraoxonase and arylesterase activities were investigated using linear regression models. The models were adjusted for age, alcohol consumption, HDL cholesterol and either waist circumference or BMI (in sensitivity analyses) as continuous variables and sex, physical activity, socioeconomic and smoking status as categorical variables. Furthermore, it was investigated whether obesity, sex or age modified the associations by applying formal tests for interaction based on a 5% significance level. For comparison, overall and sex-specific results were presented.

To improve the model fit and capture the linearity assumption between continuous covariates and the outcome, restricted cubic splines were used, if necessary. Thus, in case of non-linearity, the number of quantile-based knots was determined with respect to the adjusted R^2^, Bayesian information criterion (BIC) and likelihood ratio tests against the model with only linear terms. Multicollinearity was assessed calculating the variance inflation factor and homoscedasticity was ensured by the Breusch-Pagan test as well as visual approval of residual plots. Due to high leverage quantified by Cook’s D, one influential observation had to be removed from models with IL-6 as exposure. Finally a sensitivity analysis was done by excluding HDL cholesterol from the models. Resulting *P*-values as well as confidence intervals were corrected by the Bonferroni-Holm method. The analyses were conducted using the software R (version 4.0.3.).

## Results

### Baseline characteristics

Characteristics and circulating biomarker concentrations of the total study sample and also for men and women separately are shown in Table 1. The participants had a median age of 46 years (interquartile range (IQR) 37.8;62.0) and their median body mass index (BMI) was 25.9 kg/m^2^ (IQR 23.0;29.0). They had a median waist circumference of 92.5 cm (IQR 83.0;103.1) and a median HDL cholesterol of 45.8 mg/dl (IQR 41.2;51.8). A proportion of 10.1% belonged to the highest socioeconomic status. Furthermore, the proportions of former and current smokers were 23.4 and 24.0%, respectively; 45.6% reported being physically inactive. Median alcohol consumption was 7.5 g/day (IQR 0.4; 21.0). The median value of paraoxonase activity was 89.3 U/ml (IQR 61.1; 154.8) and of arylesterase activity was 163.7 U/ml (IQR 139.1; 189.5).

**Table 1:** Characteristics and circulating biomarker concentrations and activities in BVSII participants; median (IQR) or absolute numbers n (%)

	Overall (***n*** = 504)	Men (***n*** = 217)	Women (***n*** = 287)	***P***
Paraoxonase activity (U/ml)	89.3 (61.1; 154.8)	86.7 (60.4; 156.0)	96.5 (63.6; 154.0)	0.481
Arylesterase activity (U/ml)	163.7 (139.1; 189.5)	159.3 (138.8; 184.9)	168.3 (139.8; 194.4)	0.043
TNFα(pg/ml)	11.2 (8.8; 14.5)	12.0 (9.4; 15.3)	10.9 (8.4; 13.7)	0.004
Leptin (pg/ml)	9.3 (5.3; 16.8)	5.5 (3.5; 8.8)	13.1 (8.5; 22.7)	< 0.0001
Hs-Crp (ng/ml)	1.6 (0.8; 3.7)	1.6 (0.8; 3.4)	1.59 (0.8; 3.9)	0.807
IL-6 (pg/ml)	1.4 (0.95; 2.4)	1.5 (1.1; 2.7)	1.3 (0.9; 2.2)	0.006
Age	46.0 (37.8; 62.0)	53.0 (38.0; 64.0)	44.0 (37.0; 59.0)	0.002
Alcohol consumption (g/day)	7.5 (0.4; 21.0)	19.2 (5.1; 34.5)	3.8 (0.2; 12.2)	< 0.0001
HDL cholesterol (mg/dl)	45.8 (41.2; 51.8)	43.7 (39.9; 48.4)	48.2 (42.8; 53.5)	< 0.0001
BMI (kg/m^2^)	25.9 (23.0; 29.0)	26.6 (24.1; 29.4)	25.1 (22.3; 28.7)	< 0.0001
Waist circumference (cm)	92.5 (83.0; 103.1)	100.0 (92.5; 107.0)	86.0 (78.0; 97.3)	< 0.0001
Socioeconomic status				0.089
1 (lowest)	66 (13.1)	23 (10.6)	43 (15.0)	
2	120 (23.8)	54 (24.9)	66 (23.0)	
3	158 (31.3)	68 (31.3)	90 (31.4)	
4	109 (21.6)	42 (19.4)	67 (23.3)	
5 (highest)	51 (10.1)	30 (13.8)	21 (7.3)	
Physical activity (hours per week)				0.351
0	230 (45.6)	95 (43.8)	135 (47.0)	
0.1 to 3.9	144 (28.6)	59 (27.2)	85 (29.6)	
> =4	130 (25.8)	63 (29.0)	67 (23.3)	
Smoking status				< 0.0001
never	265 (52.6)	89 (41.0)	176 (61.3)	
former	118 (23.4)	64 (29.5)	54 (18.8)	
current	121 (24.0)	64 (29.5)	57 (19.9)	

Altogether, 43.1% of the study participants were males. In comparison to women, levels of TNF-α and IL-6 were significantly higher in men, while in women leptin levels were significantly higher than in men. Furthermore, male participants consumed significantly higher amounts of alcohol, were more often current smokers, and had a higher median BMI and waist circumference in comparison to women. Women had significantly higher HDL values and a significantly higher median arylesterase activity than men.

There were no notable correlations between inflammatory markers and paraoxonase and arylesterase activities (Spearman’s correlations: − 0.04 ≤ r ≤ 0.14).

### Regression analyses

The multivariable linear regressions were adjusted for sex (only for the total sample), age, waist circumference, physical activity, alcohol consumption, socioeconomic status, HDL cholesterol, and smoking status. They revealed consistently linear, non-significant positive (for the independent inflammatory markers TNFα and IL-6) or negative (for CRP) associations with the outcome paraoxonase activity. These relationships were found in the total sample and also in the sex-specific analyses. The results for the linear associations are shown in Table 2 (total sample) and in Table 3 (sex-specific analyses).

**Table 2:** Results of the linear regression models on the relationship between plasma inflammation markers and serum paraoxonase or arylesterase activities (total sample)

Outcome	Exposure	β (fully adjusted)^a^	95% CI corrected	***P***-value	***P***-value corrected
**Paraoxonase activity**	TNF-α	0.400	−0.407; 1.207	0.266	0.532
	Hs-CRP	−0.199	−1.206; 0.807	0.697	0.697
	IL-6	0.655	−0.367; 1.678	0.124	0.373
**Arylesterase Activity**	TNF-α	0.450	−0.156; 1.056	0.063	0.253

^a^adjusted for age, sex, waist circumference, alcohol consumption, HDL cholesterol, physical activity, socioeconomic status, and smoking status

**Table 3:** Results of the linear regression models on the relationship between plasma inflammation markers and serum paraoxonase or arylesterase activities in the sex-specific subgroup analyses

Outcome	Exposure	β (fully adjusted)^a^	95% CI	***P***-value	***P***-value corrected
**MEN**					
**Paraoxonase Activity**	TNF-α	0.624	−0.338; 1.586	0.203	1.000
	Hs-CRP	−0.177	−1.553; 1.198	0.800	1.000
	IL-6	1.844	0.504; 3.183	0.007	0.078
**Arylesterase Activity**	TNF-α	0.207	−0.442; 0.857	0.531	1.000
**WOMEN**					
**Paraoxonase Activity**	TNF-α	0.192	−0.812; 1.197	0.707	1.000
	Hs-CRP	−0.177	−1.605; 1.252	0.808	1.000
	IL-6	−0.092	−1.154; 0.971	0.865	1.000
**Arylesterase Activity**	TNF-α	0.707	0.029; 1.385	0.041	0.410

^a^adjusted for age, waist circumference, alcohol consumption, HDL cholesterol, physical activity, socioeconomic status, and smoking status

**Fig. 1:**
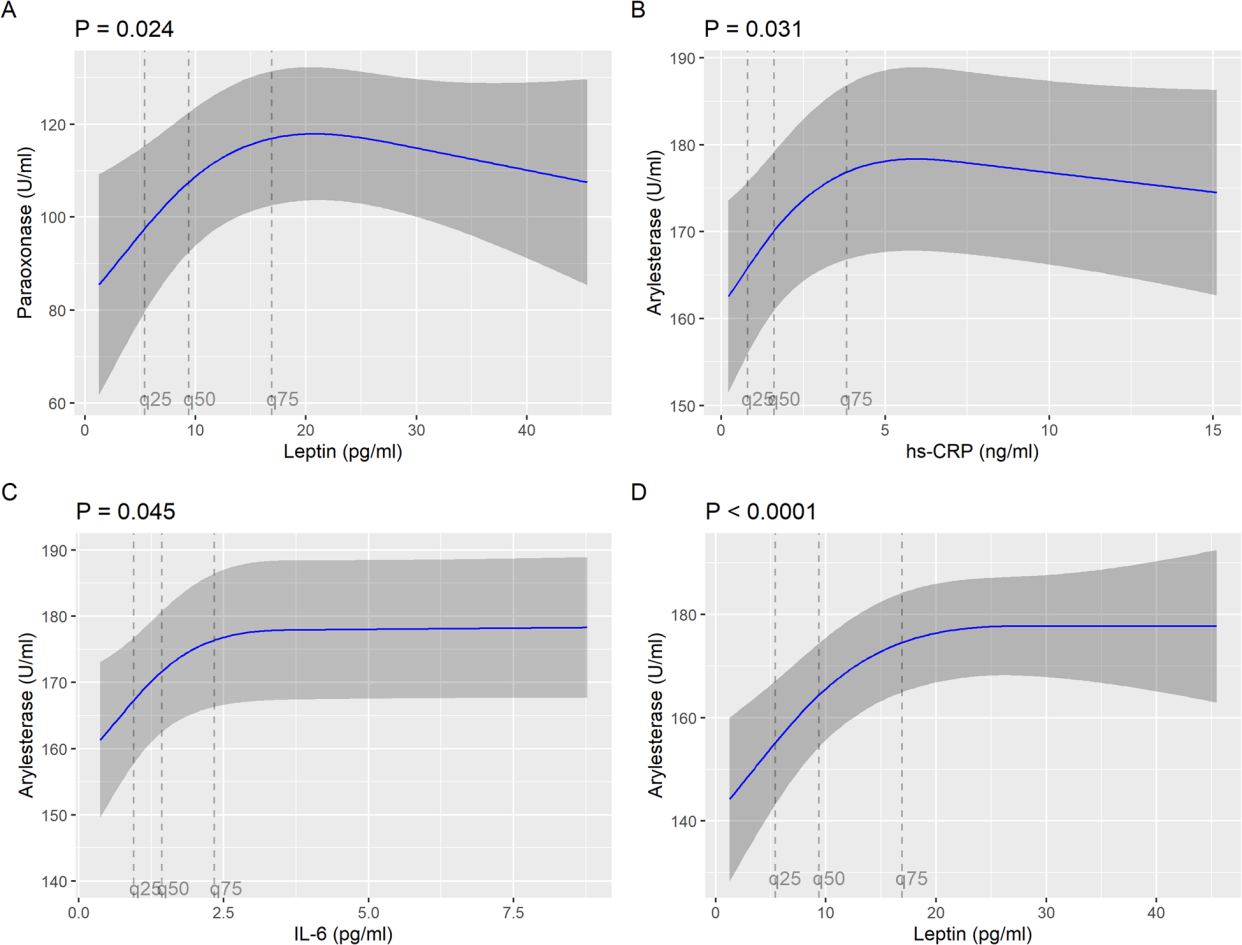
Non-linear associations between leptin, hs-CRP and IL-6 levels and paraoxonase as well as arylesterase activities, respectively (total sample). Models were adjusted for age, sex, waist circumference, alcohol consumption, HDL cholesterol, physical activity, socioeconomic status, and smoking status

**Fig. 2:**
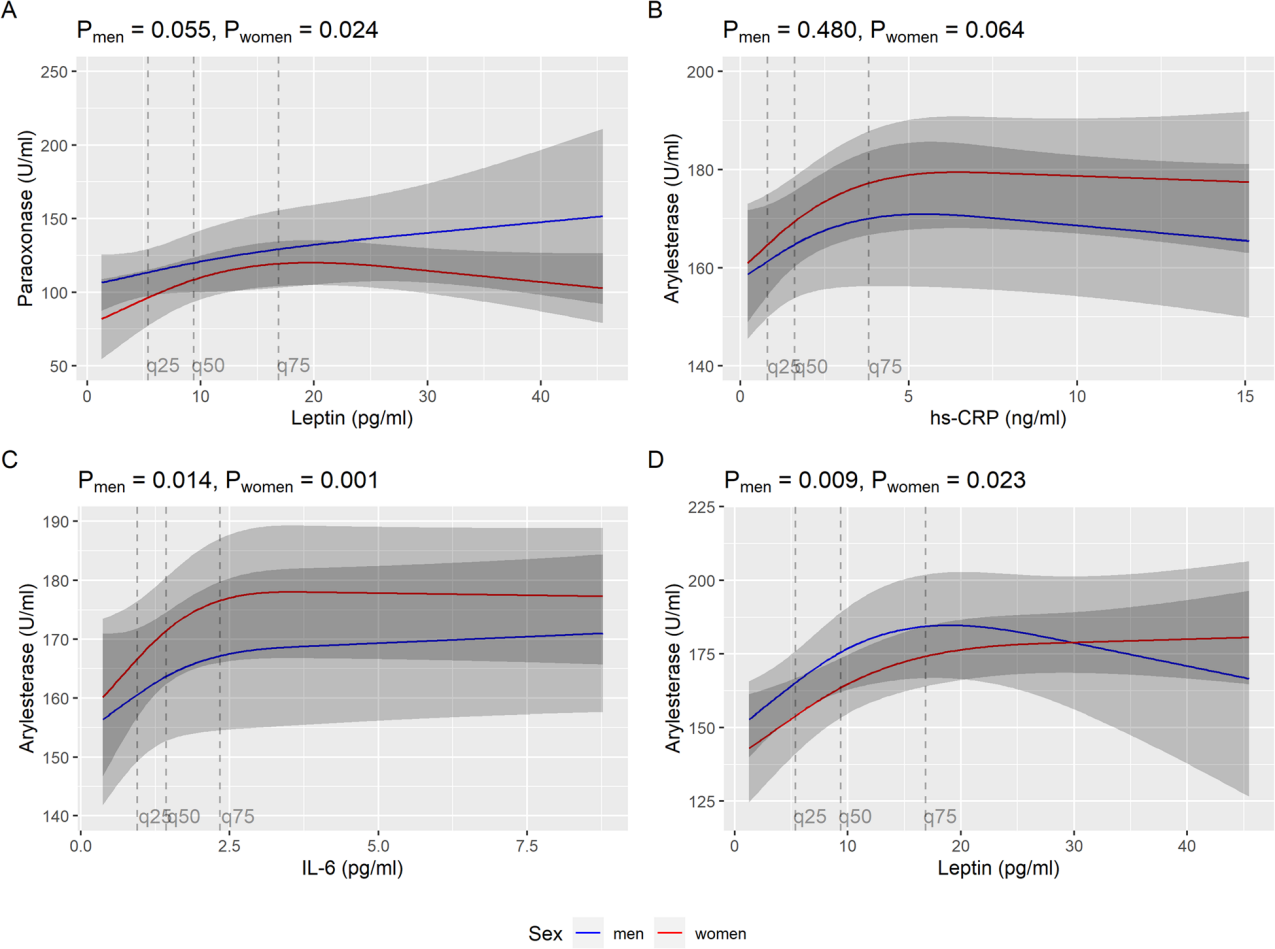
Non-linear associations in the sex-specific subgroup analyses between leptin, hs-CRP and IL-6 levels and paraoxonase as well as arylesterase activities, respectively. Models were adjusted for age, waist circumference, alcohol consumption, HDL cholesterol, physical activity, socioeconomic status, and smoking status

Leptin values were non-linearly associated with paraoxonase activity. Therefore, restricted cubic splines modelling with 3 knots was applied. The association was significant in the total sample (*P*-value for overall association 0.024), but after correction for multiple testing the significance was lost (adjusted *P*-value for overall association 0.142). Similar results were found in the sex-specific analyses. The results for the non-linear associations are displayed in Fig. 1 (total sample) and Fig. 2 (sex-specific analyses).

Multivariable linear regression models also showed a linear but non-significant positive association between TNF-α and arylesterase activity in the total sample (Table 2) as well as in the sex-specific analyses (Table 3).

Leptin as well as CRP and IL-6 were non-linearly associated with arylesterase activity in the total sample (Fig. 1). In restricted cubic spline models, the associations between CRP and arylesterase activity as well as IL-6 and arylesterase activity were significant (overall *P*-value 0.031 and 0.045, respectively), but significance did not persist after correction for multiple testing. In contrast, leptin was non-linearly but significantly associated with arylesterase activity, even after Bonferroni-Holm correction (overall *P*-value < 0.0001; adjusted overall *P*-value < 0.0001). In the sex-specific analyses these findings could be largely confirmed (Fig. 2).

### Interaction with sex, age, and obesity

In all analyses we tested for interactions between biomarkers and BMI, waist circumference, sex and age to explore whether obesity, sex or age modifies the appropriate biomarker values (data not shown). All of the tested interactions were not significant, indicating that neither obesity (*P*-value > 0.27) nor age (*P*-value > 0.06) nor sex (*P*-value > 0.19) modify the associations.

### Sensitivity analyses

In the sensitivity analyses, we adjusted the models for the aforementioned confounders but not for HDL cholesterol, and obtained quite similar results to those from the main analyses (data not shown). Furthermore, when adjusting for BMI instead of waist circumference in the multivariable analysis, comparable results could be generated (data not shown).

## Discussion

In this population-based study, circulating levels of leptin, hs-CRP and IL-6 were significantly related to arylesterase activity, which is one of the hydrolytic activities of PON1, an enzyme bound on HDL cholesterol [23]. Neither age nor sex nor obesity modified the associations. The relationship between leptin and arylesterase activity remained significant even after adjusting for multiple testing. No associations between TNF-α and paraoxonase or arylesterase activity were found.

The significant relationship between pro-inflammatory markers and arylesterase activity but not paraoxonase activity could be due to the fact that some prevalent PON1 genetic polymorphisms have almost no influence on arylesterase activity. In addition, arylesterase activity shows low interindividual variability. This is why arylesterase activity is regarded as a better surrogate of PON1 concentration than paraoxonase activity [24].

Studies examining the association between different inflammatory markers and PON1 activities in in vitro models have been performed previously. However, studies in humans are still scarce, and the existing results are inconsistent. The population-based PolSenior project, which included 3154 individuals aged 65 and higher, did investigate the association of PON1 arylestease activity with a number of factors including sex, age, several age-dependent diseases, and some serum lipids and inflammatory markers [25]. It could be shown that there was a link between lower PON1 activity and higher levels of hs-CRP and IL-6. In contrast to previous studies the present population-based study for the first time included middle-aged adults to examine whether there is an association between inflammatory markers and paraoxonase and arylesterase activities.

The cytokine IL-6 has pro- and anti-inflammatory properties [26]; it is a primary determinant of the hepatic production of CRP [27]. Levels of IL-6 are very low under normal conditions, but these levels can raise extensively as a consequence of an inflammatory response [28].

Experimental studies have shown that IL-6 and TNF-α up- and down-regulate PON1 gene expression, which suggests that inflammatory markers might have an impact on PON1 activity [29, 30]. However, whether there is a relationship between the blood levels of these inflammatory markers and PON1 levels/activity could not be answered by these studies. In the present investigation, a significant association between IL-6 (but not TNF-α) and arylesterase activities was found. Due to a lack of population-based studies on this issue, comparability with other findings is difficult.

In the BVSII also a significant association between hs-CRP, the major acute phase reactant during acute inflammation [31], and arylesterase activity was observed. Some prior studies including certain groups of patients have investigated whether high CRP levels are related to PON1 activity [14, 32, 33]. In a study by Dullaart et al. including patients with type 2 diabetes mellitus low PON1 activity was associated with higher CRP levels independent of plasma adipokines, obesity and plasma lipids [14]. In another study including persons with diabetes, persons with coronary heart disease but no diabetes, and control subjects, high CRP levels were related to low PON1 activity, suggesting that there is a mechanistic link between inflammation and the development of atherosclerosis [32]. Furthermore, a recent study including 285 hospitalized patients, 115 of whom received a surgical intervention, reported a decreased PON1 activity and an increased acute phase response in patients after surgery due to post-surgical metabolic alterations [33].

In the present study, the link between plasma leptin levels and arylesterase activity was not modified by obesity. In prior animal studies, adverse effects of leptin on PON1 activity could be shown [34, 35], and rats treated with leptin exhibited a decrease in PON1 activity [36]. However, human studies investigating such correlations at the systemic level came to different results [12, 37–40]. In a study by Bajnok et al. [12], associations of leptin with PON1 were investigated in 74 overweight-obese participants in comparison to 24 normal-weight subjects; it was reported that leptin inversely correlated with PON1, but the association was not independent from confounders [12]. Similar results were obtained in a study including obese children [38], obese females [39], and morbidly obese patients after bariatric surgery [40]. Yet contrary to these results, in a study including patients undergoing hemodialysis [37] no significant inverse association between leptin and PON1 was found. In contrast to these studies conducted in certain patient groups with rather higher (pathological) inflammatory values, the present study included mainly healthy subjects in a non-inflammatory state and in some cases persons with inflammatory values in a pathological stage. It is possible that the findings between inflammatory markers and PON1 activity in this study are due to differences between the study samples with regard to state of inflammation.

The antioxidant enzyme PON1 is mainly synthesized and secreted by the liver, and in the circulation bound to HDL transported to several tissues [41]. By binding to cell membranes, PON1 protects lipids against peroxidation [42] and prevents low-density lipoprotein oxidation [43], which is a major cause of inflammation and is involved in the initiation of inflammatory diseases such as atherosclerosis, diabetes, and cancer [44].

Thus, in the normal state, HDL has, among other features, antioxidative and anti-inflammatory properties. However, it can be hypothesized that in a state of systemic inflammation, HDL may undergo changes and lose its protective properties, possibly due to the loss of activity of the antioxidant enzyme PON1 [45]. The present study found associations between some inflammatory markers, which are correlated to cardiometabolic disease development, and arylesterase activity. The associations in the present study were generally positive until the 75th percentile and reached a plateau regarding PON1 activity with higher values of the inflammatory parameters. Interestingly, contrary to other studies reporting a relationship between inflammatory markers and PON1 in certain subgroups [12, 38], obesity, sex and age do not seem to modify the associations in this population-based sample. This is possibly due to the fact that the present study included healthy participants with mainly non-pathological levels of inflammatory markers. Further studies are needed to unravel whether and how systemic inflammation is involved in the conversion of HDL from a protective to a dysfunctional form [46–48]. Furthermore, while the activities of paraoxonase and arylesterase are now often subjects of research, the level of knowledge on lactonase activity in connection with inflammation is scarce [49]. Recent studies in certain patient groups, such as cancer patients or in patients after surgery found lower arylesterase and lactonase activity in states of chronic inflammation [50, 51]. The link between PON1 lactonase activity and inflammation should be evaluated in further studies.

### Study strength and limitations

The strengths of the present study are the large population-based sample and the availability of comprehensive data. A limitation of the study is its cross-sectional design, which cannot show causal relationships. Furthermore, the present study sample consisted of European Caucasians in a certain age-range; therefore it is not representative for other ethnicities or age-groups. Also, blood samples were collected in a non-fasting state. Prior studies could show that meal intake has an influence on the plasma concentration of inflammatory markers [52]. This fact may have influenced the findings of this study. Finally, no measurement of lactonase activity was available in the present study because knowledge on lactonase activity of PON1 was rare in 2004 and established later [53].

## Conclusions

The present study showed that plasma IL-6, hs-CRP, and leptin were non-linearly associated with arylesterase activity. Leptin levels were also significantly associated with paraoxonase activity; with increasing levels of inflammation, the activity of arylesterase and paraoxonase increased. However, with higher levels (> 75th percentile) of inflammatory parameters, the activities reached a plateau or even decreased somewhat, suggesting that such high levels of inflammation may lead to impaired enzyme activity and subsequently to dysfunctional HDL in middle-aged individuals from the general population. This finding could be clinically relevant because it suggests that this group of people may have a higher risk for cardiometabolic diseases. Whether or not a measurement of PON1 activity in combination with the lipid profile and certain inflammatory markers could improve the prediction of cardiometabolic diseases should be evaluated in clinical studies.

## Data Availability

The datasets used and analysed during the current study are available from the corresponding author on reasonable request.

## Abbreviations

BIC: Bayesian information criterion
BMI: Body mass index
BVSII: Bavarian food consumption survey II
CV: Coefficient of variation
HDL: High-densitiy lipoprotein
hs-CRP: High-sensitive C-reactive protein
IL-6: Interleukin-6
IL-1β: Interleukin-1β
IQR: Interquartile range
PON1: Paraoxonase-1
TNF-α: Tumor necrosis factor alpha
